# Measuring and monitoring patient safety in hospitals in Saudi Arabia

**DOI:** 10.1186/s12913-021-07228-z

**Published:** 2021-11-12

**Authors:** Yazeed Kaud, Sinéad Lydon, Paul O’Connor

**Affiliations:** 1 Discipline of General Practice, School of Medicine, National University of Ireland Galway, County Galway, H91 TK33 Galway, Ireland; 2 Department of Public Health, Saudi Electronic University, Riyadh, Saudi Arabia; 3 School of Medicine, National University of Ireland Galway, 1 Distillery Road, Newcastle, Co Galway, H91 TK33 Galway, Ireland

**Keywords:** Patient safety, Measurement, Monitoring, Hospital, secondary care, Saudi Arabia

## Abstract

**Background:**

There is much variability in the measurement and monitoring of patient safety across healthcare organizations. With no recognized standardized approach, this study examines how the key components outlined in Vincent et al’s Measuring and Monitoring Safety (MMS) framework can be utilized to critically appraise a healthcare safety surveillance system. The aim of this study is to use the MMS framework to evaluate the Saudi Arabian healthcare safety surveillance system for hospital care.

**Methods:**

This qualitative study consisted of two distinct phases. The first phase used document analysis to review national-level guidance relevant to measuring and monitoring safety in Saudi Arabia. The second phase consisted of semi-structured interviews with key stakeholders between May and August 2020 via a video conference call and focused on exploring their knowledge of how patient safety is measured and monitored in hospitals. The MMS framework was used to support data analysis.

**Results:**

Three documents were included for analysis and 21 semi-structured interviews were conducted with key stakeholders working in the Saudi Arabian healthcare system. A total of 39 unique methods of MMS were identified, with one method of MMS addressing two dimensions. Of these MMS methods: 10 (25 %) were concerned with past harm; 14 (35 %) were concerned with the reliability of safety critical processes, 3 (7.5 %) were concerned with sensitivity to operations, 2 (5 %) were concerned with anticipation and preparedness, and 11 (27.5 %) were concerned with integration and learning.

**Conclusions:**

The document analysis and interviews show an extensive system of MMS is in place in Saudi Arabian hospitals. The assessment of MMS offers a useful framework to help healthcare organizations and researchers to think critically about MMS, and how the data from different methods of MMS can be integrated in individual countries or health systems.

**Supplementary Information:**

The online version contains supplementary material available at 10.1186/s12913-021-07228-z.

## Introduction

Measuring and monitoring safety (MMS) is fundamental to safety improvement efforts. However, a major challenge to improving safety in healthcare is the lack of high quality information to allow organizations, teams, and individuals healthcare providers to evaluate how they are performing, and where there are deficits and risks [1]. Understanding and improving patient safety requires the availability, and application, of valid and reliable methods for measuring and monitoring safety [2]. However, MMS is not straightforward, and there is no consensus as to how it should be achieved.

The World Health Organization developed the International Classification for Patient Safety (ICPS) to provide an approach to organizing patient safety data for the purpose of aggregation, analysis, and translation into actionable information [3]. However, the ICPS is focused upon classifying and identifying the contributory factors to incidents, rather than providing a framework for evaluating a patient safety surveillance system. Recognizing that most healthcare organizations lack the capacity to analyze, monitor, or learn from safety information, Vincent et al. [4, 5] developed a framework to guide clinical teams and healthcare organizations in the measurement and monitoring of safety. This MMS framework was derived from three scoping reviews on safety measurement in high-risk industries, conceptual approaches and models of systems safety, and research on measuring safety in healthcare. Interviews were conducted with senior healthcare managers, and cases studies developed for services where measurement of safety is well developed (e.g. anesthesia) [4, 5]. This research cumulated in the identification of five safety dimensions that should be addressed by a robust safety surveillance systems [4, 5]. These dimensions are:

1. Harm: has patient care been safe in the past? (e.g. national audits, incidence of falls or pressure ulcers, mortality and morbidity).
2. Reliability of safety critical processes: are our clinical systems and processes reliable? (e.g. monitoring of vital signs, observations of safety critical behavior).
3. Sensitivity to operations: is care safe today? (e.g. safety walk-arounds and conversations, talking to patients).
4. Anticipation and preparedness: will care be safe in the future? (e.g. safety culture assessment, structured reflection).
5. Integration and learning: are we responding and improving? (e.g. aggregated analysis of incidents, feedback and implementation of safety lessons) [4, 5].

Mapping methods of MMS onto Vincent et al’s [4, 5] framework allows organizations not only to consider where safety information is lacking, but also where there is redundancy and duplication of effort. The MMS framework has been used to promote self-reflection at both board and clinical team level, to stimulate an organizational check or analysis of the gaps in safety information, and to promote discussion about safety [6]. The framework has also been applied to the classification of MMS studies in systematic reviews [2, 7]. Applying the MMS framework supports the creation of a nuanced and holistic understanding of safety, increased consciousness of safety among staff, a shared vocabulary and language around patient safety, a review of the kinds of safety data which are useful and which should be collected, and to support wider engagement in patient safety work and initiatives [6]. However, despite the potential of the MMS framework, it has not been applied to the review of a country’s healthcare safety surveillance system. Therefore, the aim of the study reported in this paper is to use the MMS framework to evaluate the Saudi Arabian healthcare safety surveillance system for hospital care.

In recent years there has been substantial investment in patient safety initiatives in the Saudi Arabian healthcare system [8]. Therefore, the aims of the current study are to: (1) examine how patient safety is measured and monitored in Saudi Arabian hospitals; (2) map the methods of MMS in these hospitals onto the five dimensions of Vincent et al’s [4, 5] MMS framework; (3) based on these findings, reflect on the approaches used to MMS in Saudi Arabian hospitals; and (4) evaluate the utility of using the Vincent et al’s [4, 5] framework to classify different methods of MMS.

## Methods

### Research design

A qualitative descriptive approach was employed to support: (1) a document analysis of the national standards on MMS used in Saudi Arabian hospitals; and (2) an exploration of stakeholders’ perceptions about how patient safety is measured and monitored in Saudi Arabian hospitals through semi-structured interviews. The research team consisted of one woman (SL) and two men (YK and POC). Two of the members of the research team (POC and SL) are PhD-level health services researchers with considerable experience in using quantitative and qualitative research methodologies. The other member of the team (YK) is a Masters-level health services researcher who was trained by POC and SL to conduct the interviews.

### Phase one-document analysis

The purpose of the first phase of the study was to identify recommended or mandated processes of MMS in Saudi Arabian hospitals. Document analysis is a systematic procedure for reviewing or evaluating documents [9]. To ensure the completeness and accuracy of the document analysis reported herein, we adhered to a method called the ‘ready materials, extract data, analyze data and distil (READ)’ approach to document analysis [10].

### Inclusion criteria

Documents were included if they: explicitly discussed or described how patient safety is, or should be, measured and monitored in Saudi Arabian hospitals; were produced by a Saudi Arabian national government agency or an organization affiliated with a national government agency, and were written in English or Arabic. To allow for retrieval of older but potentially important documents, no restrictions on publication date was specified. If a document was found to have multiple versions, only the latest version of the document was included in the review.

### Exclusion criteria

Documents were excluded if they: did not discuss how patient safety is, or should be, measured and monitored in Saudi Arabian hospitals; were not produced by a Saudi Arabian national government agency or an organization affiliated with a national government agency, or were not written in English or Arabic.

### Search process

The document search was completed in May 2020 and consisted of four steps. First, an advanced google search was completed. Second, a search of the following electronic databases was conducted: Medline, CINAHL, OAIster, IMEMR, WHO IRIS, and Google scholar using various combinations of the terms ‘measuring safety’, ‘monitoring safety’, and ‘measurement of safety’. Additional File 1 presents an exemplar search strategy. Third, searches were conducted across the Saudi Arabian Ministry of Health, Central Board for Accreditation of Healthcare Institutions (CBAHI) and Saudi Patient Safety Center websites using both their relevant search boxes and manual search. Last, we searched for further related documents by hand-searching the reference lists of documents that met the inclusion criteria.

### Document selection

The initial screening of potentially relevant documents was completed through the assessment of the titles, abstracts and/or executive summaries. Documents that appeared relevant were exported and downloaded for full-text review. Decisions regarding the inclusion or exclusion of documents were agreed by consensus of all of the members of the research team. All decisions were recorded in a Microsoft Excel© file.

### Document analysis

Two members of the research team (YK and POC) independently searched through each document and extracted all of the methods of MSS described therein. The methods of MMS extracted by each reviewer were compared. Only minor differences were found between the two reviewers. Any differences were concerned with whether a particular method was one measure or could be split into two measures. Once the final list of MMS had been identified all members of the research team (YK, POC, and SL) reviewed each measure, and reached a decision by consensus as to which dimension of Vincent et al’s [4, 5] MMS framework it addressed.

### Phase Two: Semi-structured interviews with key stakeholders

The purpose of the second phase of the study was to identify what key stakeholders know about how safety is measured and monitored in the Saudi Arabian healthcare system.

### Ethical review

 The study was approved by the Ministry of Health Central Institutional Review Board in Saudi Arabia (Central IRB log No: 20 -74E) and was performed in accordance with the Declaration of Helsinki. All participants provided signed written informed consent before participating in the study.

### Sampling and recruitment of participants

To ensure a diverse sample that represents perceptions of people in different roles in the Saudi Arabian healthcare system, participants were drawn from five different stakeholder groups: (1) policy makers; (2) doctors; (3) nurses; (4) risk managers; and (5) quality supervisors. Recruitment of participants was via a combination of purposive and snowball sampling techniques.

### Development of interview guide

The semi-structured interview guide is shown in Table 1. The structure of the interview guide was derived from the five dimensions of Vincent et al’s [4, 5] MMS framework. The questions for the interview were developed with reference to best practice for the development of interview questions [11–13]. The interview was piloted with a doctor and a policy maker. No changes were made to the guide as a result of the feedback from the pilot interviews. Therefore, these interviews were included in the study.

**Table 1:** Interview guide used to engage participants in discussion around measuring and monitoring safety in SA

1. In the Saudi healthcare system, how is harm to patients measured and monitored? 1.1. What are the strengths and limitation of methods used? 1.2. Are there other methods of measuring and monitoring harm that you think should be used? and if so, what are these and why do you think they’d be useful?	
2. What methods are in place to assess whether our clinical systems, processes and behaviour reliable? 2.1. What are the strengths and limitation of each of these methods? 2.2. Are there other methods of measuring and monitoring standardised clinical practice that you think should be used?	
3. What methods are in place to assess whether care is safe in hospitals in Saudi Arabia today? 3.1. What are the strengths and limitation of each of these methods? 3.2. Are there other methods of measuring and monitoring whether patient care is safe today you think should be used? and if so, what are these and why do you think they be useful?	
4. What methods are in place to anticipate and reduce future risks to patients’ hospitals in Saudi Arabia? 4.1. What are the strengths and limitation of each of these methods? 4.2. Are there other methods of improving the anticipation and reduction of future risk to patients that you think should be used? and if so, what are these and why do you think they be useful?	
5. What methods are in place to promote learning from issues and improving the level of patient safety in hospitals in Saudi Arabia? 5.1. What are the strengths and limitation of each of these methods? 5.2. Are there other methods of prompting learning that you think should be used, and if so, what are these and why do you think they be useful?	

### Procedure

All interviews took place between May and August 2020. After receiving signed written informed consent from all participants, the interviews were carried out by one member of the research team (YK) via a video conference call. The audio of the call was recorded.

### Interview analysis

The interview analysis was focused on identifying the methods of MMS described by the interviewees, and classifying these methods using the dimensions of Vincent et al. [4, 5] MMS framework. The transcription was carried out using Microsoft Word© by the lead author (YK) and reviewed and checked for errors by two authors (POC and SL). The methods of MMS mentioned by the interviewees were highlighted in the Microsoft Word© document by YK, and then reviewed by POC. The comment function of Microsoft Word© was then used to record the MMS framework domain identified by the researcher. All three members of the research team reviewed a random sample of five of the interview transcripts and identified and classified the MMS methods my consensus. The remaining 16 interviews were classified by consensus between two members of the research team (either YK and POC or YK and SL).

## Results

### Phase one-document analysis

**Fig. 1:**
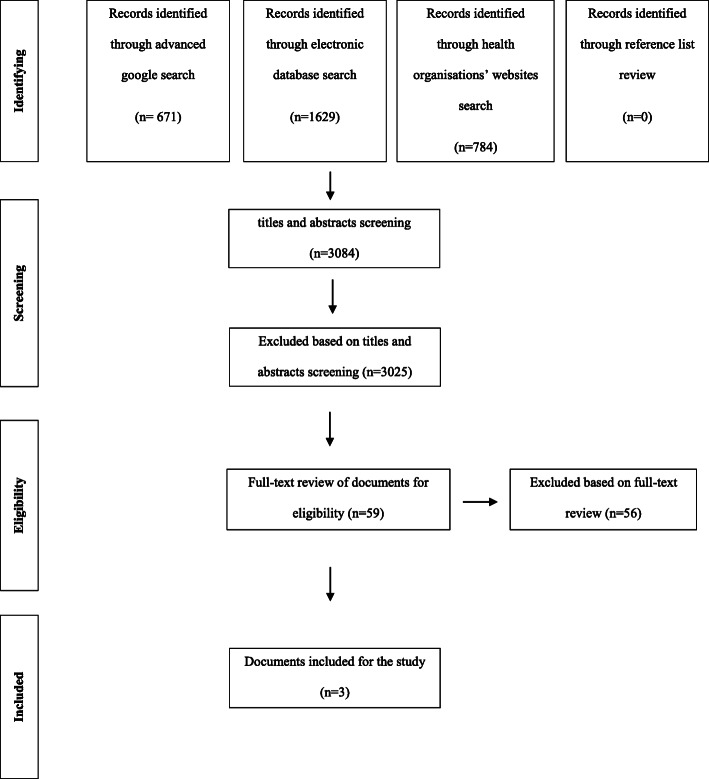
Flow diagram depicting study selection for document analysis

Figure 1 provides an overview of the search process for identifying documents that met the inclusion criteria. This process resulted in three documents. An overview of these documents is provided in Table 2. All three documents were written in English and had been published since 2015.

**Table 2:** A summary of key information of each included document

Title	CBAHI National Hospital Standards 3rd edition	Department of Quality and Safety at King Fahd Hospital of the University (KFHU) Annual Report	Quality and Patient Safety Measures in King Faisal Specialist Hospital and Research Centre (KFSH&RC)
**Year published**	2015	2015	2015
**Pages**	265	127	Virtual document
**Prepared by**	The Saudi Central Board for Accreditation of Healthcare Institutions (CBAHI)	The Directorate of Quality and Safety at KFHU	Quality Management Division (QMD) at King Faisal Specialist Hospital and Research centre
**Stated aim**	To facilitate the process of hospital self-assessment against pre-set requirements.	To highlight the DQS achievements, establish future commitments and benchmark the hospital progress.	To continuously improve the quality of care provided; maintain a risk-free environment and assure compliance with accreditation standards.
**Target population**	Hospital leadership and all HCPs working in hospitals in Saudi Arabia.	Hospital leadership and HCPs working at KFHU.	All HCPs, patients, and members of the public.
**Setting**	All healthcare services provided by all hospitals in Saudi Arabia.	All healthcare services at KFHU.	All healthcare services at King Faisal Specialist Hospital and Research Centre.
**Number of methods of MMS measures included**	35	8	3
1. Harm	9 (25 %)*	3 (37.5 %)	2 (66.7 %)
2. Reliability of safety critical processes	12 (33.3 %)	3 (37.5 %)	1 (33.3 %)
3. Sensitivity to operations	2 (5.6 %)	1 (12.5 %)	0
4. Anticipation & preparedness	2 (5.6 %)	0	0
5. Integration & learning	11 (30.6 %)	1(12.5 %)	0

HCPs: Healthcare Professionals

*One of the methods of MMS was classified under two dimensions, and so these percentages are calculated using a denominator of 36

As can be seen from Table 2, methods of MMS were far more commonly described in the Saudi Central Board for Accreditation of Healthcare Institutions (CBAHI) standards than in the other two documents, reflecting the purpose of the CBAHI to facilitate the process of hospital self-assessment against pre-set requirements [14].

A total of 39 unique methods of MMS were identified across the three documents (see Table 2 and Additional File 2 for a list of these methods and how they were classified). Of these MMS methods: 10 (25 %) were concerned with past harm; 14 (35 %) were concerned with the reliability of safety critical processes, 3 (7.5 %) were concerned with sensitivity to operations, 2 (5 %) were concerned with anticipation and preparedness, and 11 (27.5 %) were concerned with integration and learning. One methods of MMS addressed two of the safety dimensions, therefore the percentages are calculated out of 40.

### Phase Two- Semi-structured interviews with key stakeholders

The mean duration of the interviews was 34 min and 35 s (SD= 12 min 18 s) The 21 participants included: three policy makers, five doctors, seven nurses, two risk managers and four quality supervisors. Of the 21 participants, 4 were women and 17 were men. The participants reported a mean of 12.4 years of professional experience (ranging from 6 months to 35 years). Nine (14.3 %) of the participants worked in public hospitals, 3 (14.3 %) in teaching hospitals, 3 (14.3 %) in national health regulation organizations, and 2 (9.5 %) in military hospitals. None of the people interviewed were colleagues of members of the research team.

Table 3 shows the methods of MMS reported by the interviewees, with Table 4 presenting examplar quotes from the interviewees. The interviewees described a total of 37 methods of MMS (see Table 3). Of these methods of MMS, 10 (27 %) were concerned with past harm, 10 (27 %) were concerned with the reliability of safety critical processes, 3 (8.1 %) were concerned with sensitivity to operations, 7(18.9 %) were concerned with anticipation and preparedness, and 7 (18.9 %) were concerned with integration and learning.

**Table 3:** Methods reported by participants to measure and monitor patient safety in Saudi hospitals

Dimension	No.	List of reported methods of measurement	Number of participants reported the measure (no.) %		
			(12) Front-line staff	(9) Non front-line staff	(21) All
1. Harm	1	Incident reports	(7) 58.3%	(8) 88.9%	(15) 71.4%
	2	Mortality and morbidity rates	(5) 41.7%	(1) 11.1%	(6) 28.6%
	3	Patient safety indicators	(4) 33.3%	-	(4) 19%
	4	Incidence of falls	(3) 25%	-	(3) 14.3%
	5	Mortality review committees	(1) 8.3%	(1) 11.1%	(2) 9.5%
	6	Meetings and discussion of sentinel events	-	(1) 11.1%	(1) 4.8%
	7	Medication error reporting	-	(1) 11.1%	(1) 4.8%
	8	Infection rates	-	(1) 11.1%	(1) 4.8%
	9	National hotline to report safety concerns	-	(1) 11.1%	(1) 4.8%
	10	Patient satisfaction surveys	-	(1) 11.1%	(1) 4.8%
2. Reliability of safety critical processes	1	Monitoring compliance to hand hygiene	(4) 33.3%	(3) 33.3%	(7) 33.3%
	2	Observation of safety critical behaviours	(3) 25%	(2) 22.2%	(5) 23.9%
	3	Monitoring standards	-	(5) 55.5%	(5) 23.9%
	4	Reaccreditation CBAHI	(1) 8.3%	(1) 11.1%	(2) 9.5%
	5	Quality officer checks on compliance to policies and procedures	-	(2) 22.2%	(2) 9.5%
	6	Venous thromboembolism risk assessment	(1) 8.3%	-	(1) 4.8%
	7	Key performance indicators of patient safety goals	-	(1) 11.1%	(1) 4.8%
	8	Audit of equipment availability by infection control staff	(1) 8.3%	-	(1) 4.8%
	9	Infection control checklists	-	(1) 11.1%	(1) 4.8%
	10	Clinical audit	-	(1) 11.1%	(1) 4.8%
3. Sensitivity to operations	1	Safety walk-rounds	(3) 25%	(1) 11.1%	(4) 19%
	2	Ward rounds and conversations with staff	(1) 8.3%	(1) 11.1%	(2) 9.5%
	3	Talking to patients	(1) 8.3%	-	(1) 4.8%
4. Anticipation and preparedness	4	Failure mode and effect analysis (FMEA) to identify risks	-	(2) 22.2%	(2) 9.5%
	5	Staff assessment and credentialing	(2) 16.7%	(1) 11.1%	(3) 14.3%
	6	Risk registers	-	(2) 22.2%	(2) 9.5%
	7	Anticipated staffing levels	(1) 8.3%	(1) 11.1%	(2) 9.5%
	8	Hazard vulnerability analysis	(1) 8.3%	-	(1) 4.8%
	9	Safety culture assessment	-	(1) 11.1%	(1) 4.8%
	10	Systems to report near misses to identify risks	-	(1) 11.1%	(1) 4.8%
5. Integration and learning	1	Analysis and learning from incidents leading to implementation of safety lessons	(1) 8.3%	(3) 33.3%	(4) 19%
	2	Learning from root cause analysis	(1) 8.3%	(2) 22.2%	(3) 14.3%
	3	Learning and mitigation plans made based on FMEA data	-	(3) 33.3%	(3) 14.3%
	4	Feedback by clinical teams following analysis of incident reports	(1) 8.3%	(1) 11.1%	(2) 9.5%
	5	Learning from mortality and morbidity review committees	-	(1) 11.1%	(1) 4.8%
	6	Lessons following near miss reporting	-	(1) 11.1%	(1) 4.8%
	7	Sharing patient safety lessons at national level between hospitals	-	(1) 11.1%	(1) 4.8%

**Table 4:** Example quotes from the interview transcripts

Dimension	Example quotes
1. Harm	**Incident reports** “We have the incident reporting systems, you know the OVR [occurrence variance reporting], which is used in 100 % of MOH hospitals at the hospital level” (Risk Manager 2).
	**Mortality and morbidity rates** “There is the mortality rate which is reviewed monthly. If it is high, then it will be discovered as an issue and an area that requires attention” (Risk Manager 1).
	**Patient safety indicators** “Almost all hospitals tend to use patient safety indicators, and even in our hospital they tend to focus on the use of patient safety indicators” (Nurse 5).
2. Reliability of safety critical processes	**Monitoring compliance to hand hygiene** “So, if we take hand hygiene for example, we have in the emergency department a nurse whose main responsibility is to observe staff, how they are adhering to the infection control procedures, and so on (Nurse 2).
	**Observation of safety critical behaviours** “There are observations whether they are conducted by the nursing manager, medical director, or hospital director” (Risk Manager 1).
	**Monitoring standards** “The MOH constructed 3 years ago, has 20 standards that are risky, and that’s the essential safety requirements which is supervised by CBAHI. They are the main evaluator” (quality supervisor 2).
3. Sensitivity to operations	**Safety walk-rounds** “Safety walk-round involve the safety department, nursing department and the quality department” (Quality Supervisor 1).
4. Anticipation and preparedness	**Failure mode and effect analysis (FMEA) to identify risks** “Other tools we use is the FMEA [failure mode and effect analysis] and you know FMEA is one of the tools that have been used for a long time in aviation and now used in healthcare field, and it anticipates or predicts future risks to the patient or the organisation, and put solutions for these risks” (Policy Maker 2).
	**Staff assessment and credentialing** “Staff credentialing which is one of the 20 standards that is applied by MOH and we were evaluated against by CBAHI in the last three years” (Quality Supervisor 2).
5. Integration and learning	**Analysis and learning from incidents leading to implementation of safety lessons** “Sometimes the reoccurrence rates of some safety events indicate to us about the necessity to implement an intervention, a project, budget, modification or take a very quick action to resolve them” (Policy Maker 2).
	**Learning from root cause analysis** “We advise organisations to use root cause analysis because it is very intense type of analysis that leads you to the root causes of the issue and then putting action plans to prevent reoccurrence of these root causes and treat these root causes to prevent reoccurrence of these incidents” (Doctor 1).
	**Learning and mitigation plans made based on FMEA data** “In terms of FMEA, it is prospective, it is something you imagine to happen in the future, and you put the solutions as if these risks happened already, and you train and prepare people to use it. So this is considered future preparations for safety issues” (Policy Maker 2).

The most commonly described method of MMS for past harm was incident reports (mentioned by 15 interviewees; 71.4 %). Incident reports were also by far the most commonly mentioned method of MMS across all of the dimensions. The most frequently mentioned method of MMS for the reliability of safety critical processes was monitoring compliance with hand hygiene protocols (mentioned by 7; 33.3 % of the interviewees), safety walk-rounds (mentioned by 4; 19 % of the interviewees) was the most frequently described method of MMS in the sensitivity to operations dimension, staff assessment and credentialing (mentioned by 3; 14.3 % of the interviewees) was the most frequently mentioned MMS method in the anticipation and preparedness dimension, and analysis and learning from incidents (mentioned by 4; 19 % of the interviewees) was the most frequently mentioned MMS method in the integration and learning dimension.

There were differences in the level of awareness of different MMS methods between front-line staff and managers. As can be seen from Table 3, interviews that were not front-line workers were more aware of methods of MMS in the dimensions of integration and learning methods, anticipation and preparedness methods, and reliability of safety critical processes than front-line healthcare workers. However, although front-line workers were found to be less aware of the range of methods used to MMS than those in more management focused positions, front line-workers tended to blame the managers for this lack of knowledge. To illustrate, a nurse shared that *‘those who are responsible for the administration of the hospital don’t leave their offices to come and inform us why these indicators are important, why collecting such data is important. The quality meetings are only held between the managerial departments, charge nurses and other nurses are not part of these meetings. Like I said, managers and nurses don’t share the same understanding of the measures used to assess safety’* (Healthcare provider 1).

## Discussion

A major challenge to improving safety in healthcare is the lack of high quality information to allow organizations, teams, and individuals providers to evaluate how they are performing, and where there are deficits and risks. The aim of this paper was to examine how patient safety is measured and monitored in Saudi Arabia, and to evaluate the utility of using the Vincent et al. [4, 5] framework to classify different methods of MMS. The data collected through document analysis and stakeholder interviews demonstrates that there is widespread collection of safety data in Saudi Arabian hospitals, though with differences in levels of awareness across stakeholder groups, and the potential to increase data collection within some of the MMS domains. The findings also suggest that there is utility in using the MMS framework to classify methods of MMS.

Taken together, the document analysis and stakeholder interviews demonstrate widespread MMS in the Saudi Arabian healthcare system. There was coverage of each MMS domain evidenced within the data, though there was substantive variability in the extent to which data was collected within the different domains. Measures of past harm were identified most frequently (30.4 % of methods in document analysis, 27 % of methods in interviews; e.g., incident reports, incidence of falls). This is perhaps unsurprising as the assessment of past harm often forms the foundation of a healthcare organization’s safety management system, [15] and most hospital risk management systems continue to be reactive and focused on safety events which have occurred [16]. The second most common form of MMS, was the use of measures of reliability (34.8 % of methods in document analysis, 27 % of methods in interviews; e.g., monitoring hand hygiene compliance, observation of safety critical behaviors), followed by measures of integration and learning (26.1 % of methods in document analysis, 18.9 % of methods in interviews; e.g., learning from root cause analysis, lessons following near miss reporting). Measures of anticipation and preparedness (4.3 % of methods in document analysis, 18.9 % of methods in interviews; e.g., failure mode and effect analysis, safety culture assessment) and sensitivity to operations (6.5 % of methods in the document analysis and 8.1 % of methods in interviews; safety walk arounds, talking to patients) were used less frequently. This is not necessarily unexpected. Systematic reviews [2, 7] concerned with MMS in healthcare have noted measures of sensitivity to operations are used less frequently than measures within other MMS domains, and that measures of anticipation and preparedness are also used with a relatively low frequency and are typically limited to safety climate surveys. One possible explanation for the particularly infrequent use of measures of sensitivity to operations might be that measures of sensitivity to operations tend to be qualitative (e.g. talking with patients and staff and observing work). As such, it may be more challenging to capture this information than is the case for more quantitative data [17]. This type of qualitative information has been described as ‘soft intelligence’ [17, 18]. This qualitative data often escapes capture but may offer a valuable guide to potential problems [18]. Therefore, consideration should be given to how to more effectively capture this qualitative safety data within Saudi Arabian hospitals and how to triangulate this data with the more quantitative data from the other dimensions of the MMS framework.

On the whole, our data shows that there is a comprehensive system of MMS in Saudi Arabia. Engagement with MMS is crucial to facilitate identification of issues that may result in harm to patients, support implementation of effective interventions to improve patient safety, to allow for comparisons to be made between sites or even between wards within a site [4, 5]. However, it is not that more MMS is necessarily better; it has been suggested that healthcare stakeholders could get the information they need with 25 % of what is currently being spent on measurement [19] and that much mandatory MMS is excessive. Indeed, it has been suggested that in spite of the collection of a massive volume of safety-related data in hospitals, that it remains difficult to actually determine how safe care delivery is [4, 5, 20]. It is therefore important that further exploration of the various methods identified within each MMS domain is undertaken in a Saudi Arabian context through engagement with stakeholders. It is crucial to understand the value and contribution of each individual method [19], and to understand which methods should be prioritized and which are redundant [6, 21]. Which methods yield data that valuably supports learning and safety improvement? Which methods consume resources and attention without adding value to efforts to improve safety? Assessment of MMS methods in this manner, supported by engagement with stakeholders, will allow for MMS systems to be refined and optimally effective in supporting the improvement of safety in care delivery.

Some differences in the level of awareness of different MMS methods between front-line staff and those in more managerial roles emerged during the interviews. The interviewees that did not work at the front-line appeared more likely to be aware of integration and learning methods, anticipation and preparedness methods, and reliability of safety critical processes methods of MMS. This awareness may be at least partly explained by the fact that it has been traditionally the role of risk managers to identify risks and prepare response and mitigation plans [22]. However, the majority of front-line healthcare workers in our interviews expressed the belief that they should fully understand and be engaged in the risk management process. Moreover, as a result of their experience, it could be argued that front-line workers have a more valid understanding than managers who are likely to be removed from front-line operations. It is generally considered essential that managers work with front-line staff to ensure that safety data are appropriately prioritized, interpreted, and actioned [4, 5]. Greater involvement of front-line staff in MMS activities can be valuable for helping these staff members better understand and think about patient safety [4, 5], and to support realization of the value of MMS for quality and safety improvement [6]. Risk assessment techniques, such as failure mode and effect analysis (FMEA), and incident investigations, such as Root Cause Analysis, should be carried out by a multi-disciplinary team [23, 24]. Therefore, it is suggested that consideration is given as to how to involve front-line healthcare workers in MMS in ways that are valuable and sustainable, and how to simplify complex MMS methods, and their resulting data, in order to facilitate their use by busy front-line healthcare workers [22]. We have previously suggested it is important to begin to refine extensive MMS systems in place within the Saudi Arabia healthcare system in order to ensure these are maximally efficient and that resources invested yield data that will effectively support quality and safety improvement. It is imperative that such efforts are inclusive of all stakeholder groups, including managers and front-line workers.

The current paper offers a useful opportunity to reflect upon the patient safety practices and systems currently in operation in Saudi Arabia. As discussed above, the data collated herein demonstrate a high level of activity across each of the MMS domains. However, there are a number of areas that should be considered by those interested in advancing understanding of, and ability to improve, patient safety in the Saudi Arabian healthcare system. First, there was little evidence of patient involvement across the MMS methods identified within this paper, with only two methods identified that were inclusive of patients (patient satisfaction surveys, talking to patients). Patients and their families have valuable insights into the functioning of the healthcare system [25], with privileged access to information on continuity of care, communication failures, and respect issues [26]. The importance of involving patients in patient safety efforts have been well-explicated [20, 27]. There are various ways in which patients can provide data on safety. For example, through patient-report safety climate surveys [28], bedside interviews [29], in the review of their clinical notes for errors [30], systematic review of healthcare complaints [26], and patient incident reporting tools [31]. As work is undertaken to refine a MMS system in Saudi Arabia, it is crucial that methods that are inclusive of patients are incorporated. Second, it will be important to explore barriers and facilitators to particular MMS methods, or the domains of MMS measurement. Such data would valuably support refinement of the MMS system. However, the focus of our interviews was on the identification of methods rather than considering the value, feasibility, or attitudes towards these as perceived by different stakeholder groups. It is well established in the research literature that there are barriers associated with the implementation of different MMS methods [32–34] or systems and with patient safety interventions [35, 36]. Indeed, there is some data emerging from Saudi Arabia relating to barriers to the use of incident reporting systems [37, 38]. Understanding barriers and facilitators associated with various processes is key to supporting effective implementation [39]. Therefore, while a relatively extensive system of MMS is in operation in Saudi Arabia, it will be important to understand engagement with, and attitudes towards, different methods of MMS. This will facilitate the effective use of individual methods and may also support refinement of an overall system of MMS. Finally, when considering how the existing MMS systems in Saudi Arabian hospitals may be refined it will be important to understand the value of the data coming from individual methods. As we have noted previously, it has been argued that much mandatory MMS is excessive and/or redundant [19]. It is important to understand what MMS methods yield data which can be actioned to support quality and safety improvement. Safety interventions can come from use of the MMS framework but this is not guaranteed and there is a baseline level of knowledge required, including expertise in improvement, to support its effective use [6]. There is a recognized need to improve quality of care in Saudi Arabia [40], including the safety of care. This is coupled with an increased focus on quality improvement in Ministry of Health hospitals [41]. It is important that there is engagement with those involved in implementing quality improvement endeavors to understand what data is being used to support these, the value of data arising from each MMS method, and perceived data gaps pertaining to safety. This work is particularly important given that participants were least likely to identify methods within the MMS Integration and Learning domain in this study’s interviews.

The MMS framework was developed to provide a conceptual model to guide organizations in assessing safety [6]. It was not specifically designed to classify the methods used to MMS- although this is how we have used it in this paper, as well as in two previous systematic reviews [2, 7]. As the MMS framework was not developed for classifying methods of MMS, the dimensions are not completely operationalized, and it could be argued that the dimensions are not entirely mutually exclusive- properties that are desirable for a classification system [42]. Therefore, although we found the MMS framework to be useful, there were some challenges in using it to classify methods of MMS. It is suggested that if the MMS framework is to be used for classification, then consideration should be given on how to operationalise the dimensions and clarification is required in order to specify which method of MMS correspond to which dimension. Vincent et al. [4, 5] provide some specific examples of methods of MMS, and the dimension that they address. However, if the MMS framework is to be used as a classification system it would be beneficial if there was an exhaustive list of methods of MMS that had been classified using the five dimensions from the framework. This classification is something that could be carried out by a group of subject matter experts and then shared. Pre-classification would greatly simplify the MMS mapping process, support the consistency and reliability of the classification, and facilitate (inter)national comparison across healthcare organizations. Finally, an exhaustive list of pre-classified methods of MMS would also provide examples of methods that could be added to a safety surveillance system if deficiencies in a particular safety dimension were identified.

## Limitations

There are a number of limitations of this research. The main limitation is that this paper only focused on MMS in the Saudi Healthcare system. However, the study offers a framework or process that could be replicated in other countries to support improvement of MMS processes. Only a small number of documents met the inclusion criteria and the study provides focused insights from a small number documents and a limited number of interviews. In common with other qualitative research approaches, this study could be critiqued due to subjectivity in the reporting and accuracy of the data, and the analysis. In order to address these potential issues, a rigorous approach was taken to the data collection and analysis. Second, only national-level documents were examined, which may have resulted in the exclusion of potentially useful hospital-level documents. However, the difficulty in systematically accessing hospital-level documents precluded their inclusion. Finally, the study only focused on the Saudi Arabian healthcare system. Therefore, this limits the generalizability of the findings. Nevertheless, it is suggested that there is merit in carrying out a similar exercise in other national healthcare systems in order to reflect on how safety is being measured and monitored.

## Conclusions

Although there is no single perfect method of MMS, data from a large number of measures can be challenging to interpret, and lead to confusion about how safety can be improved. The document analysis and interviews conducted in the current study show an extensive system of MMS is in place in Saudi Arabia hospitals. Going forward it will be important to engage all stakeholder groups in order to refine and optimize the system for MMS to ensure it is capable of effectively supporting safety improvement. The assessment of MMS undertaken in the current study may offer a useful framework that will help healthcare organizations and researchers internationally to think critically about MMS, and how the data from different methods of MMS can be integrated in individual countries or health systems. Such thinking will support the design of a safety surveillance system that has the range of measures require to support an understanding of what is being done well, where improvements are required, and whether interventions having the desired effect.

## Supplementary information

**Additional file 1**

**Additional file 2**

## Data Availability

All data arising from the document analysis is either presented in the article or within the included supplemental material. Interview data is summarized within the article. The ethical approval for this study does not include provision for sharing interview transcripts.
