# Supplementary material for: Measuring and monitoring patient safety in hospitals in Saudi Arabia
Source: BMC Health Serv Res. 2021 Nov 12;21:1224. doi: 10.1186/s12913-021-07228-z (PMC8588732; doi:10.1186/s12913-021-07228-z)
Supplement: Supplementary file 2 — Additional file 2 [file 12913_2021_7228_MOESM2_ESM.docx]

**Additional File 2**

**Document analysis**

1. Harm- have we been safe in the past?

2. Reliability of safety critical processes

3. Sensitivity to operations- are we safe today?

4. Anticipation and preparedness- will we be safe in the future?

5. Integration and learning- are we responding and improving?

| **Measure** | **Dimension** |
| --- | --- |
| **Quality and Patient Safety Measures in King Faisal Specialist Hospital and Research Centre (KFSH&RC) (2015)** | |
| Hand hygiene audit | 2 |
| Number of patient falls that result in injury | 1 |
| Number of hospital acquired pressure injuries | 1 |
| **Department of Quality and Safety at King Fahd Hospital of the University (KFHU) Annual Report (2015)** | |
| P10. Conducted a patient record review | 1 |
| P11. Tracer activities- assessed compliance with standards by ‘tracing’ the care experiences that a patient had received to assess standards compliance | 2 |
| P12. Leadership safety walk arounds- leaders go to the wards/units and talk to front line staff about safety. | 3 |
| P13. Monitoring of KPIs (e.g. patient falls). | 1 |
| P14. Use of occurrence variance reporting system in order to monitor any event or circumstance not consistent with the standard routine operations of the hospital. | 2 |
| P15. Conducted environmental safety monitoring to measure the effectiveness of safety protocols against agreed standards of high quality and safety | 2 |
| P109. Collect adverse event reports. | 1 |
| P109. Analyse adverse event reports | 5 |
| **CBAHI National Hospital Standards 3^rd^ edition (2015)** | |
| P17. Accreditation survey- a broad assessment using multiple methods of data collection (e.g. observations, interviews, document review) in order to determine the hospital’s compliance and performance. Three days, if failed must develop a corrective action plan. Planned and random surveys | 2 |
| P32. Reporting of sentinel events | 1 |
| P33. Learning from sentinel events using root cause analysis to re-design processes and systems. | 5 |
| P48. Inspections of medical supplies and devices | 2 |
| P48. Reporting of adverse events with medical devices | 2 |
| P55 Conduct staff satisfaction surveys. | 1 |
| P61 Conduct monthly morbidity and mortality meetings to review relevant cases, and; | 1 |
| P61 make recommendations for actions for improvement based on M and M meetings | 5 |
| P61 Medical record review committee monitors the documentation in medical records for quality, completeness, and timeliness. | 2 |
| P61 Medical record review committee makes recommendations for improvement as required | 5 |
| P63. Utilization committee assesses the appropriateness of care, e.g. quality of care | 2 |
| P63 Utilization committee make recommendations for actions for improvement | 5 |
| P64. Operating room, tissue, pharmacy and therapeutic committee monitors performance. | 2 |
| P64. Operating room, tissue, pharmacy and therapeutic committee makes improvements in the relevant areas. | 5 |
| P64. Cardiopulmonary resuscitation committee reviews all codes. | 2 |
| P64. Cardiopulmonary resuscitation committee identifies actions for improvement. | 5 |
| P75 Regular evaluation of the activities and outcomes of the rapid response teams (team that responds to a rapidly deteriorating patient outside ICU). | 2 |
| P88/89. Analysis of indicators such as mortality rates, healthcare associated infections, patient satisfaction, adverse events, medication errors, sentinel events, patient complaints. | 1,5 |
| P88/89. The hospital monitors its performance through regular data collection | 2 |
| P88/89. Regularly collected data is used to make improvements. | 5 |
| P.90 Systematic process is used to identify and analyse potential risks for severity and likelihood of occurrence e.g. use of proactive approaches such as failure mode and effects analysis | 4 |
| P103. The hospital collates trended data on patient complaints. | 4 |
| P103. The hospital takes a QI and strategic actions based on trended data on patient complaints. | 1 |
| P180. The hospital has a system to monitor the completeness of medical records. | 2 |
| P184. There is continuous surveillance of healthcare associated infections. | 2 |
| P185 Results of surveillance of healthcare associated infections are integrated into QI programme. | 5 |
| P192. Compliance with hand hygiene is regularly monitored. | 2 |
| P199 Monitor and evaluate adverse drug events. | 1 |
| P212. The hospital conducts analysis of all adverse drug reactions. | 1 |
| P212. Hospital collects data on significant and potential medication errors | 1 |
| P212. Hospital conducts root-cause analysis for significant and potential medication errors, and uses this data to change practices | 5 |
| P212. Healthcare professionals receive feedback on medication errors and near misses. | 5 |
| P227 System for reporting adverse blood donation events. | 1 |
| P227 System for monitoring adverse blood donation events. | 3 |
| P239 Safety committee conducts quarterly and as needed safety tours to identify risks and hazards related to facilities and physical plant and evaluates staff knowledge. | 3 |
